# Maternal exposure to ambient air pollution and risk of congenital heart defects in Suzhou, China

**DOI:** 10.3389/fpubh.2022.1017644

**Published:** 2023-01-04

**Authors:** Li Sun, Qianlan Wu, Huiying Wang, Juning Liu, Yan Shao, Rong Xu, Tian Gong, Xiaoju Peng, Baoli Zhang

**Affiliations:** Suzhou Maternal and Child Healthcare Center, Suzhou Municipal Hospital, The Affiliated Suzhou Hospital of Nanjing Medical University, Suzhou, China

**Keywords:** ambient air pollution, gestational exposure, birth defects, congenital heart defects, Suzhou

## Abstract

**Background:**

More and more studies have investigated the association between maternal exposure to ambient air pollution during pregnancy and incidence of congenital heart defects (CHDs), but results are controversial. The aim of this study was to investigate whether maternal exposure to air pollutants (PM_10_, PM_2.5_, NO_2_, CO, SO_2_) are associated with an increased risk of congenital heart defects in Suzhou city, China.

**Methods:**

Based on the birth defect monitoring system of Suzhou city and the Environmental Health Department of Suzhou CDC, the birth defect monitoring data and concentrations of five air pollutants (PM_10_, PM_2.5_, NO_2_, CO, SO_2_) in Suzhou city from 2015 to 2019 were obtained. The distribution of demographic characteristics of children with birth defects and exposure to air pollutant concentrations during different pregnancy periods were analyzed, Chi-square test was used to analyze whether there were statistical differences in the distribution of parturient woman age, pregnant weeks, times of pregnancy, as well as fetal sex and birth weight among children with congenital heart defects and other defects. Logistic regression model was further established to calculate the adjusted odds ratios (aORs) and 95% confidence intervals (CI) for the association between exposure to these ambient air pollutants during pregnancy and CHDs.

**Results:**

A total of 5,213 infants with birth defects were recruited in this study from 2015 to 2019, the top five birth defects in Suzhou were syndactyly, congenital heart disease, ear malformation, cleft lip and palate, and hypospadias, and the proportion of congenital heart disease increased. The level of maternal exposures (mean ± sd) was highest in first trimester amongst pregnant women in Suzhou city. Compared to other birth defects, we observed significant increasing associations between PM_2.5_ exposure during second and third trimester with risk of CHDs, aORs were 1.228 and 1.236 (95% CI: 1.141–1.322, 1.154–1.324 separately) per a 10 μg/m^3^ change in PM_2.5_ concentration. Maternal NO_2_ exposure was significantly associated with CHDs in first trimester (aOR = 1.318; 95% CI: 1.210–1.435).

**Conclusions:**

Our study contributes to explore the current state of Suzhou air quality and the association between maternal air pollution exposure and congenital heart defects. Exposure to PM_2.5_ and NO_2_ is thought to increase the risk of CHDs, but comprehensive description of these associations will be needed in future studies.

## 1. Introduction

Congenital heart defects (CHDs) is a serious structural abnormality of the large blood vessels in the heart or chest cavity. It is one of the most common types of birth defects in the world, as well as the leading cause of infant death and physical disability, which has a significant impact on individuals, families and society (1). There were an estimated 900,000 new birth defects are born in China every year and the overall incidence of birth defects is about 5.6%, which is similar to moderately developed countries. According to national maternal and child health monitoring data, the incidence of CHDs increased from 11.40 per 10,000 in 2000 to 126.62 per 10,000 in 2019, showing an upward trend during this period (2). The incidence in Suzhou is consistent with the national trend during recent 4 years. In 2020, the incidence of congenital heart disease in Suzhou is 2,642/10,000, accounting for 25.3% of total perinatal birth defects and recognized to be major cause of neonatal death due to birth defects.

Environmental pollutants have been considered as one of the important causes of congenital malformations. The role of prenatal exposure to ambient air pollution on birth defects has gradually become a research hotspot (3). “Barker hypothesis” once pointed out that gestation is a critical period in determining on later susceptibility to certain chronic conditions. Developing embryos are sensitive to environmental pollutants because of undergoing rapid cell proliferation and differentiation (4). It has been shown that air pollutants can cause adverse pregnancy outcomes by interfering with cellular endocrine function, oxidative stress, inflammation, DNA damage, and other pathways (5). Dozens of studies revealed that atmospheric pollutants (such as O_3_, CO, SO_2_, NO_2_, PM) may contribute individually or in combination to adverse pregnancy outcomes like low birth weight (LBW), preterm birth (PTD) and congenital abnormalities of small gestational age (SGA) (6, 7). Previous researches mostly focused on impacts of exposure to ambient air pollution during pregnancy on premature birth and low birth weight, the evidence for birth defects was much less explored. Moreover, studies on the relationship between ambient air pollution and CHDs were inconsistent, as well as the timing of susceptibility during pregnancy was unclear. Several epidemiological studies and meta-analyses have reported that *in utero* exposure to ambient air pollution potentially increase the risk of congenital heart defects in newborns (8, 9). In contrast, some scholars demonstrated that ambient air pollution has little effects on birth defects and even has a protective effect (10–12). Therefore, much more attention should be paid on exposure of ambient air pollution in pregnant women. To the best of our knowledge, most previous studies assigned exposure using daily pollutant averages during weeks 3–8 of pregnancy of pregnancy. Some researchers expanded the observation window to the first trimester of gestation as this is the critical period of gestation associated with congenital anomalies. Nevertheless, these methods did not capture the temporal variability in exposure across specific windows with cardiac development, which could mask or attenuate associations. In this study, we have evaluated associations between maternal exposure to air pollution and congenital heart defects at the trimester-specific scale to analyze the susceptibility window period. Moreover, very few studies have been to explore the associations between PM2.5 and congenital heart defects in developing countries, which may have higher pollution levels. So far, there were only a few studies in China. Consequently, the association between absorbable particulate matter and congenital heart defects warrants further investigation. Considering the expected rise in air pollutant levels associated with urbanization and industrialization in Suzhou. This study included the data of birth defects and air pollutants from 2015 to 2019 to investigate whether gestational exposure to ambient air pollution is associated with an increased risk of congenital heart disease among the population of Suzhou during the second and third periods as well as the first period. The following pollutants were examined: nitrogen dioxide (NO_2_), sulfur dioxide (SO_2_), carbon monoxide (CO), particulate matter with aerodynamic diameter smaller than 10 μm (PM_10_) and 2.5 μm (PM_2.5_). In addition, we estimated the timing of susceptibility during pregnancy.

## 2. Methods

### 2.1. Study population

Our data on birth defects were extracted from the birth defect monitoring sub-module system in the Suzhou Maternal and Child Health Monitoring Manual during 2015 to 2019. This includes records from perinatal babies born between 28 weeks' gestation and 7 days after birth (including live births and stillbirths), reported by midwifery agencies. The diagnosis of Birth Defects was performed by qualified doctors of medical institutions based on the “International Statistical Classification of Diseases and Related Health Problems, Tenth Edition” (ICD-10). For neonates classified as “congenital heart defects to be confirmed”, we followed up until 3 months after birth and made a supplementary report according to the follow-up results to avoid omissions. “Birth Defects Registration Card” was used to collect information including date of birth, gestational age, infant sex, maternal age, gravidity, and parity. The data included mothers living in Suzhou during pregnancy, according to maternal residential address. Excluding pregnant women whose permanent residence outside Suzhou in the Maternal and Child Health Monitoring Manual, a total of 5,213 infants with birth defects were recruited in this study, of which 1,039 infants were diagnosed with CHDs. This study was approved by the Ethics committee of Suzhou Municipal Hospital (approval No. K-2022-020-K01). In the final database, only mothers and newborns numbers were listed. All personal information was kept confidential.

### 2.2. Exposure assessment

#### 2.2.1. Exposure time window

We divided the entire pregnancy into three trimesters. Daily exposures were averaged over three exposure windows: the first trimester (1–13 + 6 weeks gestation), the second trimester (14–27 + 6 weeks gestation), the third trimester (28 weeks to delivery) (13).

#### 2.2.2. Exposure to ambient air pollutants

The ambient air pollution data collected in this study came from the Environmental Health Department of Suzhou CDC. Monitored pollutants including nitrogen dioxide (NO2), sulfur dioxide (SO2), carbon monoxide (CO), particulate matter with aerodynamic diameter smaller than 10 μm (PM10) and 2.5 μm (PM2.5) in Suzhou city. Daily 24–h average concentrations of these pollutants were obtained from 11 national air quality monitoring stations and 11 provincial air quality monitoring stations during 2014–2019 that included the period of maternal pregnancy in the present study. These air quality monitoring stations are evenly distributed in 10 districts and counties in Suzhou, including two in Zhangjiagang, two in Changshu, two in Taicang, three in Kunshan, three in Wujiang, two in Wuzhong, two in Xiangcheng, one in Gusu, two in Suzhou industrial park, and three in Suzhou National Hi-Tech District.

Firstly, the average daily concentration was calculated by the obtained daily concentrations from the 22 monitoring stations. Exposure during the first, second and third trimesters was, respectively, calculated as the average of the monthly mean concentrations of NO2, SO2, CO,PM10, and PM2.5 during the first to third month, the fourth to sixth month, and the seventh to the last month of pregnancy. The concentrations of the pollutants were expressed as μg/m^3^.

### 2.3. Statistical analysis

The database was analyzed using SPSS 17.0 software (Chicago Illinois, USA). Potential confounding variables are obtained from the “Birth Defects Registration Card” including gestational age, infant sex, maternal age, gravidity, and parity. The date of conception was defined as the 14th day after the date of the last menstruation, which was classified into spring (March to May), summer (June to August), autumn (September to November), and winter (December to February) according to the climate characteristics in the city. Categorical variables are presented as numbers and frequencies while continuous variables are represented by mean ± standard deviation. Distributions of ambient air pollutant concentrations were presented by quartile and interquartile range (IQR) averaged during different trimester of pregnancy. Pearson correlation between exposures to air pollutants and between trimesters was analyzed. Chi-square test was performed to examine statistically significant differences in social demographic characteristics between infants with CHDs and infants with other BDs. *P* < 0.05 was considered statistically significant.

We used multiple logistic regression models to evaluate the association between maternal exposure to ambient air pollution during different trimesters and risk of congenital heart defects by adjusting for potential confounding covariates. CHDs was the dependent variable, and the individual exposure concentration of air pollutants during different trimesters of pregnancy were the independent variables. Exposure variables were included in the models as continuous variables, with separate models for each pollutant. Multi-pollutant models were further performed to adjust the effects of other air pollutants. Corresponding odds ratio (OR) and 95% confidence interval (95% CI) were calculated after important covariates were controlled. Results are presented as the change in outcome every 10 mg/m^3^ increase in concentration ultimately for each air pollutant.

### 2.4. Quality controls

All deliveries were examined by a physician after birth and a professional doctor or nurse at each registered hospital was required to complete the Birth Defects Registration Card. Quality control measures were monitored regularly in respective hospitals, monthly at the county level and every half year at the municipal level in order to ensure the authenticity of the reported information. The quality requirements for birth defect monitoring data included: 100% completion rate of form, form items error rate < 1%, input error rate < 1%, and a rate of missed birth defects < 1%.

## 3. Results

Table 1 shows the distribution of birth defect types in Suzhou from 2015 to 2019. The top five birth defects were syndactyly, congenital heart defects, ear malformation, cleft lip and palate, hypospadias in this time. The proportion of congenital heart disease in total defects increased in the past 2 years, and it became the most common birth defect in 2019.

**Table 1:** Distribution of birth defect types in Suzhou from 2015 to 2019.

**Year**	**Total defects**	**Syndactyly**		**Congenital heart defects**		**Ear malformation**		**Cleft lip and palate**		**Hypospadias**	
	***n***	***n***	**Proportion (%)**	***n***	**Proportion (%)**	***n***	**Proportion (%)**	***n***	**Proportion (%)**	***n***	**Proportion (%)**
2015	1,121	209	18.6	179	16.0	180	16.1	66	5.9	23	2.1
2016	1,063	182	17.1	121	11.4	220	20.7	56	5.3	27	2.5
2017	839	227	27.1	132	15.7	176	21.0	73	8.7	25	3.0
2018	990	319	32.2	250	25.3	147	14.8	58	5.9	38	3.8
2019	1,200	308	25.7	357	29.8	170	14.2	74	6.2	60	5.0
Total	5,213	1,245	23.9	1,039	19.9	893	17.1	327	6.3	173	3.3

There were statistically significant differences in the distribution of maternal age, number of pregnancies, as well as infant gender and birth weight among children with congenital heart defects and others (*P* < 0.05). There were no statistically differences for total previous live births and season of conception, which were no longer included in the Logistic regression model as shown in Table 2.

**Table 2:** Distribution of demographic characteristics of children with birth defects.

**Characteristics**		**Congenital heart defects**		***χ^2^***	***P*-value**

		**Yes**	**No**		
		***n*** **(%)**	***n*** **(%)**		
Infant gender	Male	532 (51.2)	2,610 (62.5)	49.488	< 0.001^*^
	Female	507 (48.8)	1,551 (37.2)		
	Unknown gender	0	13 (0.3)		
Birth weight (g)	< 2,500 g	231 (22.2)	517 (12.4)	69.472	< 0.001^*^
	2,500–4,000 g	732 (70.5)	3,390 (81.2)		
	≥4,000 g	76 (7.3)	267 (6.4)		
Number of pregnancies	1	403 (38.8)	1,475 (35.3)	10.028	0.007^*^
	2–3	479 (46.1)	2,151 (51.5)		
	≥4	157 (15.1)	548 (13.1)		
Total previous live births	1	580 (55.8)	2,231 (53.4)	1.885	0.170
	≥2	459 (44.2)	1,943 (46.6)		
Season of conception	Spring	245 (23.6)	992 (23.8)	1.023	0.796
	Summer	257 (24.7)	1,089 (26.1)		
	Autumn	194 (18.7)	748 (17.9)		
	Winter	343 (33.0)	1,345 (32.2)		
Maternal age	< 20	37 (3.6)	222 (5.3)	9.583	0.008^*^
	20–35	873 (84.0)	3,532 (84.6)		
	≥35	129 (12.4)	420 (10.1)		

* *p* < 0.05.

Descriptive statistics of maternal ambient air pollution exposure during different gestation are shown in Table 3. The median range of PM_10_, PM_2.5_, NO_2_, CO, SO_2_ was 70.44–72.98, 44.27–48.49, 46.77–48.76, 828.41–871.14, 13.68–14.82 μg/m^3^. Individual exposure to each pollutant (mean ± SD) was highest in the first trimester of pregnancy. The daily average concentrations of PM_10_, PM_2.5_, NO_2_, CO, SO_2_ were 71.88, 48.79, 49.11, 849.62, 15.61 μg/m^3^, respectively. Table 4 shows pearson correlation between exposures to air pollutants during different gestation. The pollutants during each trimester were strongly correlated with each other, ranged from 0.676 to 0.961. Therefore, the risk of each pollutant could be further evaluated by using multi-pollutant model to adjust the effects of other pollutants.

**Table 3:** Maternal exposure level of each pollutant during different gestation (μg/m^3^).

	**Pollutant**	**Mean ±SD**	**Minimum**	**Maximum**	***P*_25_**	***P*_50_**	***P*_75_**
First trimester	PM_10_	71.88 ± 18.43	36.74	127.93	57.37	72.07	83.42
	PM_2.5_	48.79 ± 17.44	19.45	96.26	33.98	48.05	59.49
	NO_2_	49.11 ± 12.35	25.38	80.60	40.20	47.31	57.72
	CO	849.62 ± 211.80	388.10	1,309.43	738.10	871.14	1,000.00
	SO_2_	15.61 ± 7.87	3.69	43.64	9.17	14.56	19.36
Second trimester	PM_10_	71.24 ± 16.34	37.67	109.49	59.28	72.98	81.00
	PM_2.5_	47.88 ± 15.72	21.40	84.53	34.83	48.19	57.66
	NO_2_	48.84 ± 10.67	28.03	73.45	40.81	48.76	56.71
	CO	831.27 ± 209.66	397.96	1,186.38	701.02	864.22	1,007.24
	SO_2_	15.05 ± 7.40	4.16	35.96	8.30	14.82	19.43
Third trimester	PM_10_	69.55 ± 17.31	25.00	135.14	55.44	70.44	80.21
	PM_2.5_	46.10 ± 16.35	11.57	113.43	31.89	44.27	56.49
	NO_2_	47.68 ± 11.27	19.43	97.86	39.55	46.77	56.17

**Table 4:** Pearson correlation between exposures to air pollutants during different gestation.

**Air pollutants**		**PM_10_**	**PM_2.5_**	**NO_2_**	**CO**	**SO_2_**
First trimester	PM_10_	1	0.938^*^	0.871^*^	0.676^*^	0.756^*^
	PM_2.5_		1	0.849^*^	0.690^*^	0.755^*^
	NO_2_			1	0.705^*^	0.745
	CO				1	0.797^*^
	SO_2_					1
Second trimester	PM_10_	1	0.961^*^	0.908^*^	0.752^*^	0.790^*^
	PM_2.5_		1	0.914^*^	0.720^*^	0.792^*^
	NO_2_			1	0.762^*^	0.786^*^
	CO				1	0.823^*^
	SO_2_					1
Third trimester	PM_10_	1	0.944^*^	0.881^*^	0.729^*^	0.754^*^
	PM_2.5_		1	0.887^*^	0.711^*^	0.761^*^
	NO_2_			1	0.714^*^	0.714^*^
	CO				1	0.829^*^
	SO_2_					1

* Correlation is significant at the 0.05 level (2-tailed).

Table 5 shows the adjusted odds ratios for CDs associated with air pollutants during different gestation after adjustment for all the potential covariates and the effects of other air pollutants in the same time window. The susceptible windows of air pollutants were different. For PM_2.5_, we observed a significant association with BDs for every 10 μg /m^3^ increase in concentration particularly in the second (aOR = 1.228; 95% CI: 1.141–1.322) and third (aOR = 1.236; 95% CI: 1.154–1.324) trimester of pregnancy. But the risk of congenital heart disease was 1.318 (95% CI: 1.210–1.435) for every 10 μg/m^3^ increase in NO_2_ in the first trimester. We did not observe significant association between PM10 and CHDs. More specifically, CO and SO_2_ were significant negatively correlated to CHDs in all trimesters of pregnancy.

**Table 5:** Multivariate logistic regression analysis of air pollutants and congenital heart disease in different gestation.

	**Pollutant**	***P*-value**	**OR (95% CI)**
First trimester	NO_2_	< 0.001^*^	1.318 (1.210–1.435)
	CO	0.001^*^	0.991 (0.985–0.996)
	SO_2_	< 0.001^*^	0.609 (0.515–0.720)
Second trimester	PM_2.5_	< 0.001^*^	1.228 (1.141–1.322)
	CO	< 0.001^*^	0.988 (0.982–0.994)
	SO_2_	< 0.001^*^	0.607 (0.498–0.741)
Third trimester	PM_2.5_	< 0.001^*^	1.236 (1.154–1.324)
	CO	< 0.001^*^	0.983 (0.977–0.990)
	SO_2_	< 0.001^*^	0.671 (0.550–0.819)

* Adjustment for all the potential covariates in Table 2 [infant gender, birth weight (g), number of pregnancies, maternal age] and for the effects of other air pollutants in the same time window.

## 4. Discussion

Due to the effective implementation of prevention measures for primary and secondary birth defects, perinatal neural tube defects, congenital hydrocephalus and other fatal or severely disabling birth defects have continued to decline, and are no longer the main types of birth defects. Our results showed that congenital heart disease has become the major birth defect in recent years. One of the most likely reasons is that the congenital heart disease screening program for children aged 0–3 years was carried out as a government free practical project in Suzhou. The expansion of the screening population for congenital heart disease has resulted in an increase in the detection rate of mild congenital heart disease.

With the rapid development of urbanization and industrialization, most parts of China are suffering from serious air pollution in the past few decades. Suzhou is located in central Yangtze River Delta, suffering from severe air-pollution in recent years due to fast growing economic development. The “2016 Report on the State of the Environment in China” revealed that air quality index (AQI) of Suzhou ranked 44th in 74 survey cities. It has gradually become an important public health problem that cannot be ignored. Some scholars have observed that alveolar ventilation rate of pregnant women increased and the air pollutants inhaled may be much higher than those non-pregnant women (14). As a result, fetuses are at increased risk of exposure to contaminants. Epidemiological studies reported that prenatal exposure to air pollutants has a direct negative impact on birth outcomes (15, 16). The specific pathogenesis of adverse pregnancy outcomes caused by maternal exposure to environmental pollutants is still unclear, which may be closely related to placental dysfunction, abnormal HPO axis, and other genotoxic damage. To clarify the risk of ambient air pollution on CHDs has certain reference significance for pregnant women.

In this study, we observed that maternal exposure to PM_2.5_ increased the risk of CHDs. However, the effect might be influenced by the period of exposure during pregnancy. Existing studies have generally agreed that maternal exposure to environmental pollutants is one of the high risk factors for premature and low birth weight infants (3). There are few studies on adverse outcomes including stillbirth, abortion, and congenital defects, and the epidemiological evidence for association with congenital heart disease remains limited and inconsistent. The inconsistencies in these findings may be attributed to the heterogeneity of sources, components and exposure levels of environmental pollutants, statistical methods and population differences in demography, topography, meteorology, socioeconomic status, and personal lifestyle, as well as the role of unknown confounding factors.

Most early studies measured the average level of pollutants within 3–8 weeks after pregnancy to determine the association between exposure and the incidence of congenital heart disease when analyzing the susceptibility window period of exposure to environmental pollutants during pregnancy (10, 11, 17–19). It is well-known that 2–8 weeks is a critical period for the formation of fetal heart structure, the heart begins to develop as early as the 3rd week of gestation, and the development of the fetus's four-chamber heart forms at the 7–8th week of gestation (20). Subsequently, more and more scholars have noticed that the susceptibility window of air pollution may not be completely consistent with the life cycle of heart development. They are no longer limited to measuring the exposure within 3–8 weeks of gestation, and divide pregnancy into longer exposure periods, such as early, middle, late trimester or gestational month (12, 21–23). A population-based longitudinal case-control study in China found that exposure to higher levels of air pollutants during the first trimester was associated with an increased risk of coronary heart disease in offspring (24). In this study, average daily exposure values were calculated during the first, middle and late trimesters to expand the observation window. The results showed that the risk of congenital heart defects was 1.027 (95% CI: 1.019–1.036) for every 1 μg/m^3^ increase in NO_2_ in the first trimester, and the risks of congenital heart disease were 1.013 (95% CI: 1.001–1.025) and 1.022 (95% CI: 1.015–1.029), for every 1 μg/m^3^ increase in PM_2.5_ in the second and third trimester, respectively. Our studies thus revealed that adverse effects of environmental pollutants may have cumulative effects, and the susceptibility window may not be directly consistent with the critical stage of fetal heart development. Findings from a study in Hefei also supported this hypothesis, researchers assessed the effect of weekly air pollutants exposure on CHDs to fully reflect the sensitivity of the specific window, results showed that exposure to air pollutants (SO_2_, NO_2_, PM_10_, and PM_2.5_) increase the risk of CHDs and the main susceptible exposure windows are second and third trimester of pregnancy (25). However, no association between maternal PM_10_ exposure and CHDs was observed in our study, which is similar to the findings of some studies (21). Possibly because of larger particles (PM_10_) deposit more in the upper bronchus, while smaller particles (PM_2.5_) deposit more in the deep lung with a higher surface area to mass ratio, which may lead to enhanced toxicity.

Some studies even observed an inverse association between maternal PM_2.5_ exposure and CHDs when the type of congenital heart defects was subdivided. The results of National Birth Defects Prevention Study (NBDPS) in the United States revealed that exposure to fine particulate matter was positively associated with hypoplastic left heart syndrome (HLHS) and negatively associated with atrial septal defects (11). Vinikoor-Imler et al. also reported an inverse association between fine particulate matter exposure and atrial septal defect (ASD) (26). Studies in Australia and California have found reduced odds of ventricular septal defects with increasing prenatal exposure to environmental pollutants during pregnancy (12, 27), but none of these studies found an association between other CHDs types and environmental pollutants. It is similar to our study about CO and SO_2_ has a protective effect on CHDs. “Air pollution paradox” has been proposed to explain the correlation, which could lead to an increase early spontaneous abortion then in turn contribute to an inverse correlation in epidemiological studies.

Our study has several strengths. To the best of our knowledge, this is the first study to investigate the association between maternal exposure to air pollutants and congenital heart defects in Suzhou. In the study, the unified standard was adopted to diagnose congenital heart disease, and the Municipal maternal and child healthcare institutions carried out quality control every quarter to ensure that there would be no under-reporting or conceal-reporting, which ensured the authenticity, accuracy and completeness of reported defect cases. For neonates classified as “congenital heart defects to be confirmed”, we followed up until 3 months after birth and made a supplementary report according to the follow-up results to avoid omissions. However, there are still several limitations in this study. First, the assessment of air pollutant exposure level adopts the average value of the whole city due to we did not have data for distance between where pregnant women live and work during pregnancy and the nearest air quality monitoring stations, which is not accurate enough. Second, mobility during the gestational is not considered, which may lead to the misclassification of exposure. However, Zhang et al. revealed that only 2.6% of pregnant women changed residence during pregnancy (21). Third, data on pregnancy termination before 28 weeks of gestation were not included in this study, which may lead to underestimation of the occurrence of congenital heart disease. Finally, although several relevant covariates were controlled for in this study, we cannot eliminate the possibility of residual confounding from other factors, such as e genetic problems of parents and fetal, maternal complications, passive smoking, and time spent outdoors. Maternal smoking and alcohol use were not controlled for because < 0.3% of the mothers during pregnancy reported smoking or drinking alcohol (22).

In conclusion, our study showed that exposure to NO_2_ in the first trimester and PM_2.5_ in the second and third trimester were associated with an increased risk of congenital heart disease in offspring with birth defects. Well-designed cohort studies and more detailed environmental pollutant exposure assessment methods are necessary to validate our findings.

## Data availability statement

The raw data supporting the conclusions of this article will be made available by the authors, without undue reservation.

## Ethics statement

This study was approved by the Ethics Committee of Suzhou Municipal Hospital (approval No. K-2022-020-K01). Written informed consent from the participants was not required for this study in accordance with the local legislation and institutional requirements.

## Author contributions

LS: collecting data, data analysis, paper writing, and quality control. QW: collecting data and quality control. HW, JL, and YS: collecting data. RX, TG, XP, and BZ: data analysis. All authors contributed to the article and approved the submitted version.
